# Effects of an intense, high-frequency laser field on bound states in Ga_1 − *x*_In_*x*_N_*y*_As_1 − *y*_/GaAs double quantum well

**DOI:** 10.1186/1556-276X-7-606

**Published:** 2012-10-31

**Authors:** Fatih Ungan, Unal Yesilgul, Serpil Şakiroğlu, Esin Kasapoglu, Ayse Erol, Mehmet Cetin Arikan, Huseyin Sarı, Ismail Sökmen

**Affiliations:** 1 Physics Department, Cumhuriyet University, Sivas, 58140, Turkey; 2 Physics Department, Dokuz Eylül University, İzmir, 35140, Turkey; 3 Physics Department, İstanbul University, İstanbul, 34459, Turkey

**Keywords:** Double quantum well, Intense laser field, Dilute nitride

## Abstract

Within the envelope function approach and the effective-mass approximation, we have investigated theoretically the effect of an intense, high-frequency laser field on the bound states in a Ga_*x*_In_1 − *x*_N_*y*_As_1 − *y*_/GaAs double quantum well for different nitrogen and indium mole concentrations. The laser-dressed potential, bound states, and squared wave functions related to these bound states in Ga_1 − *x*_In_*x*_N_*y*_As_1 − *y*_/GaAs double quantum well are investigated as a function of the position and laser-dressing parameter. Our numerical results show that both intense laser field and nitrogen (indium) incorporation into the GaInNAs have strong influences on carrier localization.

## Review

### Background

Recently, the evolution of the growth techniques such as molecular beam epitaxy and metal-organic chemical vapor deposition combined with the use of the modulation-doped technique made it possible the fabrication of low-dimensional heterostructures such as single and multiple quantum wells, quantum wires, and quantum dots. In these systems, the restriction on the motion of the charge carriers allows us to control the physical properties of the structures. The studies on these systems offer a wide range of potential applications in the development of semiconductor optoelectronic devices [1-5].

GaInNAs/GaAs quantum well (QW) lasers have been attracting significant scientific interest mainly due to their applications in 1.3- or 1.55-μm optical fiber communication [6-12]. These lasers are predominantly based on GaInAsP alloys on the InP substrates, which have a higher temperature sensitivity compared to shorter wavelength lasers that are grown on GaAs substrates. The high-temperature sensitivity is primarily due to Auger recombination and the weak electron confinement resulting from the small conduction band offset in the GaInAsP/InP material system. GaInNAs alloys grown on GaAs substrates have been proposed as a possible alternative to the GaInAsP/InP system for achieving lasers with high-temperature performance [13]. The deeper conduction band well and the larger electron effective mass will provide better confinement for electrons and better match of the valence and conduction band densities of state, which leads to a higher characteristic temperature and higher operating temperature, higher efficiency, and higher output power [6-13].

As known, high-frequency intense laser field (ILF) considerably affects the optical and electronic properties of semiconductors [14-20]. Because when an electronic system is irradiated by ILF, the potential of the system is modified which affects significantly the bound state energy levels, a feature that has been observed in transition energy experiments. The design of new efficient optoelectronic devices depends on the understanding on the basic physics involved in this interaction process. Thus, the effects of a high-frequency ILF on the confining potential and the corresponding bound state energy levels are a very important problem. This problem has been a subject of great interest, and an enormous amount of literature has been devoted to this field [21-27]. However, up to now, to the best of our knowledge, no theoretical studies have been focused on the bound states in Ga_1 − *x*_In_*x*_N_*y*_As_1 − *y*_/GaAs double quantum well (DQW) under the ILF.

The purpose of this work is to investigate the effect of ILF, nitrogen (N), and indium (In) mole fractions on the bound states in Ga_1 − *x*_In_*x*_N_*y*_As_1 − *y*_/GaAsDQW. The paper is organized as follows: in the ‘Theoretical overview’ section, the essential theoretical background is described. The next section is the ‘Results and discussion’ section, and finally, our calculations are given in the ‘Conclusions’ section.

#### Theoretical overview

The method of approach used in the present study is based on non-perturbation theory developed to describe the atomic behavior under intense, high-frequency laser field conditions [28,29]. It starts from the space-translated version of the semi-classical Schrödinger equation for a particle moving under the combined forces of potential and a radiation field derived by Kramers in the general context of quantum electrodynamics [30]. For simplicity, we assume that the radiation field can be represented by a monochromatic plane wave of frequency *ω*. For linear polarization, the vector potential of the field in the laboratory frame is given by $A{(\text{t) = A}}_{0}\cos\left(\omega t\right)\hat{e}$, where $\hat{e}$ is the unit vector. By applying the time-dependent translation $r\;=r\;+\alpha \left(t\right)$, the semi-classical Schrödinger equation in the momentum gauge, describing the interaction dynamics in the laboratory frame of reference, was transformed by Kramers as follows [30]:

(1) $$-\frac{\hbar^{2}}{2m^{*}}\nabla^{2}\varphi \left(r,t\right)+V\left(r+\alpha \left(t\right)\right)\varphi \left(r,t\right)=i\hbar \frac{\partial \varphi \left(r,t\right)}{\partial t}\text{,}$$

where *V*(**r**) is the atomic binding potential, and

(2) $$\alpha (\text{t)=}\alpha_{0}\sin\left(\omega t\right)\hat{e},\;\alpha_{0}=\frac{eA_{0}}{m^{*}c\omega }$$

represents the quiver motion of a classical electron in the laser field, and *V*(**r** + *α*(*t*)) is the ‘dressed’ potential energy. In this approximation, the influence of the high-frequency laser field is entirely determined by the ‘dressed potential’ *V*(**r** + *α*(*t*)) [30],

(3) $$\;\alpha_{0}=\left(\frac{I^{1/2}}{\omega^{2}}\right)\left(e/m^{*}\right){\left(8\pi /c\right)}^{1/2}\text{,}$$

where *e* and m* are absolute value of the electric charge and effective mass of an electron; *c*, the velocity of the light; *A*_0_, the amplitude of the vector potential; and *I*, the intensity of ILF.

Following the Floquet approach [29,30], the space-translated version of the Schrödinger equation, Equation 1, can be cast in equivalent form of a system of coupled time independent differential equations for the Floquet components of the wave function *φ*, containing the (in general complex) quasi-energy *E*. An iteration scheme was developed to solve this; for the zeroth Floquet component *α*_0_, the system reduces to the following time-independent Schrödinger equation [29-32]:

(4) $$\left[-\frac{\hbar^{2}}{2m^{*}}\nabla^{2}+V\left(r,\alpha_{0}\right)\right]\varphi_{0}=E\varphi_{0}\text{,}$$

where *V*(**r**, *α*_0_) is the dressed confinement potential which depends on *ω* and *I* only through *α*_0_[28].

By applying the above-described dressed potential theory to our particular Ga_1 - x_In_x_N_y_As_1 - y_/GaAs DQW system, we write down the time-independent Schrödinger equation in one-dimensional case for an electron inside a Ga_1 - x_In_x_N_y_As_1 - y_/GaAs DQW (we choose the *z*-axis along the growth direction) in the presence of an intense high-frequency laser field (the laser-field polarization is along the growth direction), which is given by the following:

(5) $$-\frac{\hbar^{2}}{2m^{*}}\frac{\partial^{2}\psi \left(z\right)}{\partial z^{2}}+V\left(\alpha_{0},z\right)\psi \left(z\right)=E\psi \left(z\right)\text{,}$$

where *ψ*(*z*) is the wave function, and *V*(*α*_0_, *z*) is the dressed confinement potential which is given by the following expression:

(6) $$\begin{array}{l} V\left(\alpha_{0},z\right)=V_{0}\left[\theta \left(-\alpha_{0}-L/2-z\right)\right]\\ \;+\frac{V_{0}}{\pi }\left[\Theta \left(z+\alpha_{0}+L/2\right)\theta \left(\alpha_{0}-L/2-z\right)\right]\\ \;\times arc\cos\left[\frac{z+L/2}{\alpha_{0}}\right]+\\ V_{0}\left[\theta \left(-\alpha_{0}-L/2+z\right)\right]+\frac{V_{0}}{\pi }\left[\Theta \left(-z+\alpha_{0}+L/2\right)\right)\\ \;\left(\theta \left(\alpha_{0}-L/2+z\right)\right]\\ \;\times arc\cos\left[\frac{L/2-z}{\alpha_{0}}\right]+\\ V_{0}\left[\Theta \left(\alpha_{0}+L_{b}/2+z\right)-\theta \left(z-\alpha_{0}\right)\right]-\\ \frac{V_{0}}{\pi }\left[\begin{array}{l} \Theta \left(z+\alpha_{0}+L_{b}/2\right)\theta \left(-z+\alpha_{0}-L_{b}/2\right)\\ \;\times arc\cos\left[\frac{z+L_{b}/2}{\alpha_{0}}\right]+\\ \Theta \left(-z+\alpha_{0}\right)\theta \left(z+\alpha_{0}\right)\times arc\cos\left[\frac{-z}{\alpha_{0}}\right] \end{array}\right]+\;\\ V_{0}\left[\Theta \left(\alpha_{0}+L_{b}/2-z\right)-\theta \left(-z-\alpha_{0}\right)\right]-\\ \frac{V_{0}}{\pi }\left[\begin{array}{l} \Theta \left(-z+\alpha_{0}+L_{b}/2\right)\theta \left(z+\alpha_{0}-L_{b}/2\right)\\ \;\times arc\cos\left[\frac{-z+L_{b}/2}{\alpha_{0}}\right]+\\ \Theta \left(-z+\alpha_{0}\right)\theta \left(z+\alpha_{0}\right)\times arc\cos\left[\frac{z}{\alpha_{0}}\right] \end{array}\right] \end{array}$$

where *V*_0_ is the conduction band offset at the interface; *L* = *Lw*_1_ + *Lw*_2_ + *L*_*b*_, *Lw*_1_ = *Lw*_2_, the well width; *L*_*b*_, the barrier width; Θ, the Heaviside unit step function which satisfies *Θ*(*z*) = 1 − *θ*(−*z*); and *θ*, the unit step function [33].

To solve the Schrödinger equation in Equation 5, we take as base the eigenfunction of the infinite potential well with *L*_*s*_ width. *L*_*s*_ is the well width of the infinite well at the far end of DQW with *L* width (*L*_*s*_ > > *L*), and its value is determined according to the convergence of the energy eigenvalues. These bases are formed as [34] follows:

(7) $$\psi_{n}\left(z\right)=\sqrt{\frac{2}{L_{s}}}\cos\left[\frac{n\pi }{L_{s}}z-\delta_{n}\right]\text{,}$$

where

$$\delta_{n}=\{\begin{array}{l} 0\;\text{if}\;n\;\text{is odd,}\\ \frac{\pi }{2}\;\text{if}\;n\;\text{is even,} \end{array}$$

and so, the wave function in the *z*-direction is expanded in a set of basis function as follows:

(8) $$\psi \left(z\right)=\sum_{n=1}^{\infty }c_{n}\psi_{n}\left(z\right)\text{.}$$

In calculating the wave function *ψ*(*z*), we ensured that the eigenvalues are independent of the chosen infinite potential well width *L*_*s*_ and that the wave functions are localized in the well region of interest. This method, which gives accuracies greater than 0.001 meV, is well controlled, gives the DQW eigenfunctions, and is easily applied to situations of varying potential and effective mass.

### Results and discussion

In this work, we have theoretically investigated the effects of ILF, In, and N concentrations on the bound states in Ga_1 - x_In_x_N_y_As_1 - y_/GaAs DQW. The energy levels and corresponding wave functions of an electron confined in the Ga_1 - x_In_x_N_y_As_1 - y_/GaAs DQW under the ILF are calculated within the framework of the effective mass and envelope-wave function approximation. The band structure parameters used in this study are from [35,36]. The bandgap energy and electron effective mass of Ga_1 - x_In_x_N_y_As_1 - y_/GaAs is calculated using the band-anti-crossing model (BAC). The electron effective mass of Ga_1 - x_In_x_N_y_As_1 - y_/GaAs as predicted by BAC model is given by [37,38]:

(9) $$\begin{array}{l} m^{*}\left(Ga_{1-x}In_{x}N_{y}As_{1-y}\right)=2m^{*}\left(In_{x}Ga_{1-x}As\right)/\\ \;\times \left(1-\frac{E_{C}-E_{N}}{\sqrt{{\left(E_{C}-E_{N}\right)}^{2}+4V_{\mathrm{NC}}^{2}y}}\right)\text{.} \end{array}$$

The *E*_−_ in the BAC model is taken to be the fundamental bandgap energy (*E*_*G*_) for Ga_1 - x_In_x_N_y_As_1 - y_,

(10) $$E_{-}=\frac{1}{2}\left[\left(E_{N}+E_{C}\right)-\sqrt{{\left(E_{N}-E_{C}\right)}^{2}+4V_{\mathrm{NC}}^{2}y}\right]\text{,}$$

(11) $$E_{C}=E_{C0}-1.55y$$

(12) $$E_{N}=1.65\left(1-x\right)+1.44x-0.38x\left(1-x\right)$$

(13) $$V_{\mathrm{NC}}=2.7\left(1-x\right)+2x-3.5x\left(1-x\right)\text{,}$$

where *x* and *y* are the In and N compositions in Ga_1 − *x*_In_*x*_N_*y*_As_1 − *y*_, respectively; *E*_*C*0_, the energy in the absence of N; and *E*_*C*_, *E*_*N*_, and *V*_*NC*_, the bandgap energies of InGaAs at *Γ* point, the energy of the isolated N level in the InGaAs host material, and the coefficient describing the coupling strength between *E*_*N*_ and the InGaAs conduction band, respectively.

**Figure 1:**
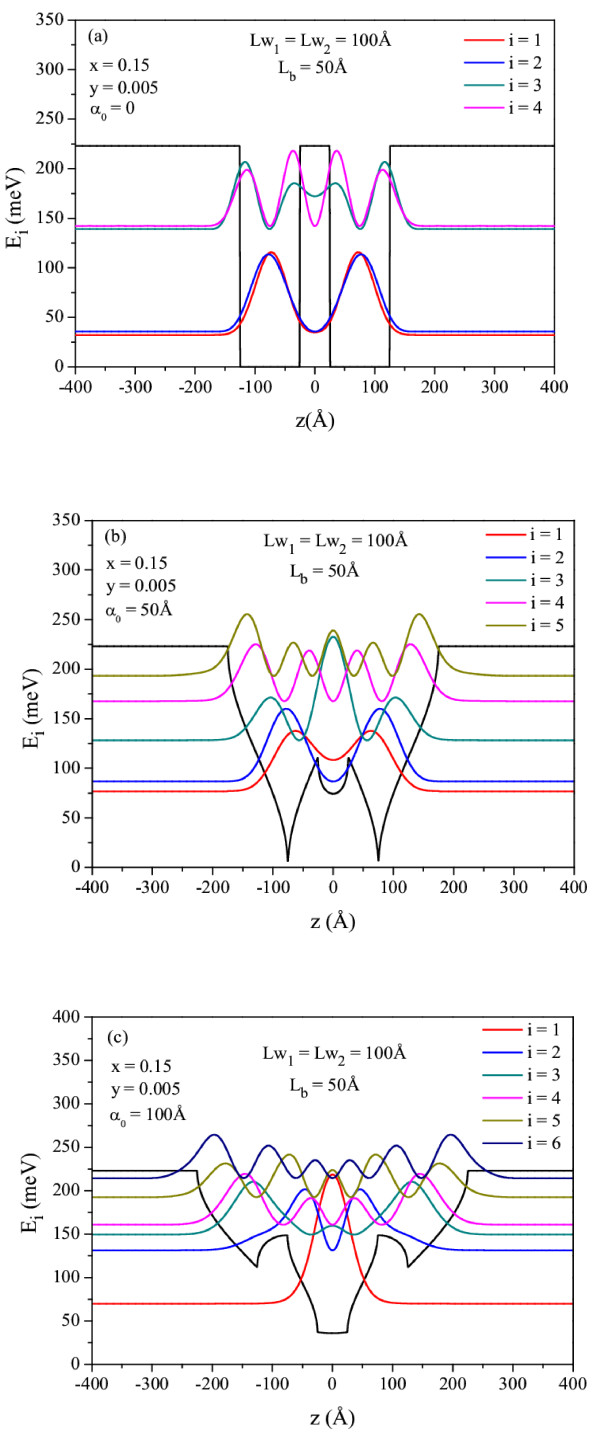
**The variation of the laser-dressed potential, bound states, and squared wave functions.** Related to these bound states in Ga_1 − *x*_In_*x*_N_*y*_As_1 − *y*_/GaAs DQW which has the width *L*w_1_ = *L*w_2_ = 100 Å, *L*_b_ = 50 Å as a function of the position. In and N concentrations are *x* = 0.15, *y* = 0.005, respectively. The results are as follows: (**a**) *α*_0_ = 0 Å, (**b**) *α*_0_ = 50 Å, and (**c**) *α*_0_ = 100 Å.

In Figure 1a,b,c, we show the laser-dressed potential, bound states, and squared wave functions related to these bound states in Ga_1 − *x*_In_*x*_N_*y*_As_1 − *y*_/GaAs DQW which has the width *L*w_1_ = *L*w_2_ = 100 Å, *L*_b_ = 50 Å for a constant In and N concentrations *x* = 0.15, *y* = 0.005, and different laser-dressing parameters (*α*_0_) as a function of the position. As seen in this figure, there are four bound states in DQW for *α*_0_ = 0 Å, while there are five and six bound states for *α*_0_ = 50 Å and *α*_0_ = 100 Å, respectively. Because as ILF increases, the width of the well bottom decreases by *L*w − 2*α*_0_, while the top width increases by *L*w + 2*α*_0_. In the meantime, the opposite behavior takes place in the barrier region. Energy levels are closer to each other since ILF creates an additional geometric confinement on the electronic states in the DQW. Furthermore, for *α*_0_ values which satisfy the condition *α*_0_ ≥ *L*w/2, the role exchange between the well and the barrier emerges: the barrier region turns into well, and the well region turns into barrier. Thus, DQW potential turns into triple quantum well as *α*_0_ increases. The emergence of role exchange between the well and the barrier opens the possibility of creating controllable resonant states located in the material. This obviously does not need any growth of conventional triple QWs, which are more difficult to tune to the desired resonance states.

**Figure 2:**
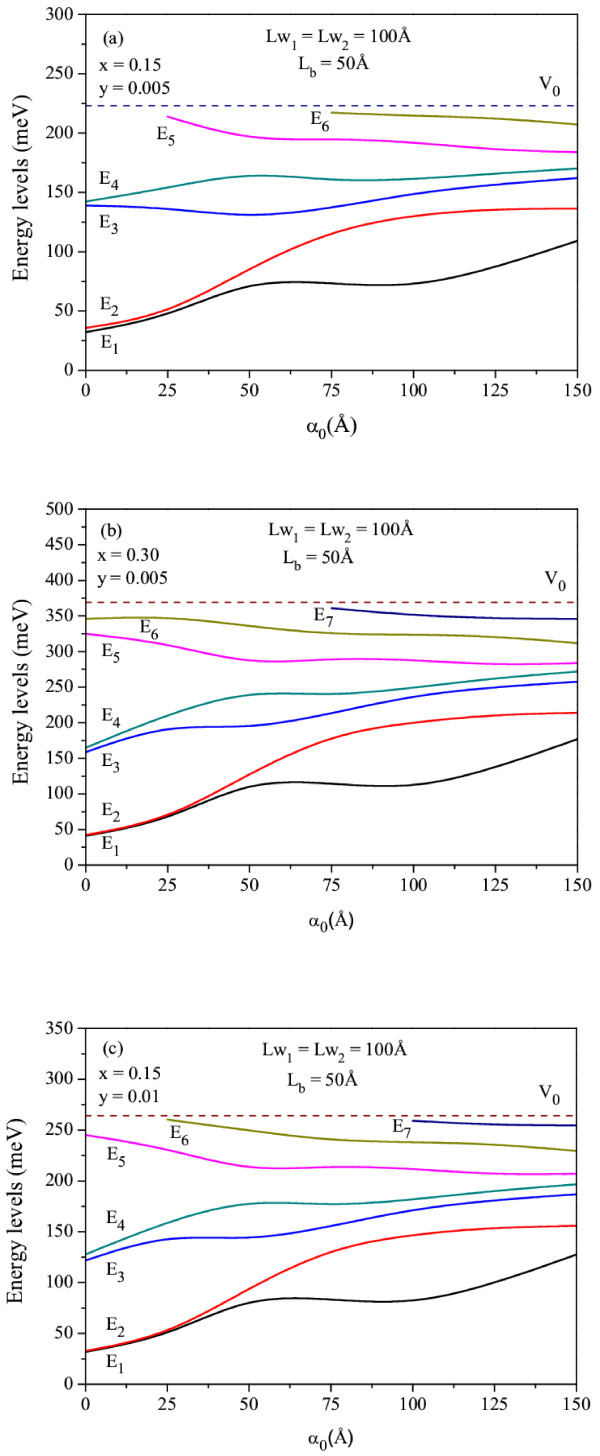
**The variation of energy levels.** For bound states in Ga_1 − *x*_In_*x*_N_*y*_As_1 − *y*_/GaAsDQW which has the width *L*w_1_ = *L*w_2_ = 100 Å, *L*_b_ = 50 Å as a function of the laser-dressing parameter. The results are as follows: (**a**) *x* = 0.15, *y* = 0.005; (**b**) *x* = 0.30, *y* = 0.005; and (**c**) *x* = 0.15, *y* = 0.01.

In order to see the effect of the ILF on the electronic states, the variations of energy levels for bound states in Ga_1 − *x*_In_*x*_N_*y*_As_1 − *y*_/GaAs DQW as a function of the laser-dressing parameter for a constant N (In) concentration and two different In (N) concentrations are given in Figure 2a,b,c, respectively. As seen in this figure, as *α*_0_ increases, the lowest energy levels increase while the bound state energies which are newly appeared with the effect of ILF decrease, and this can be appreciated as an important factor in forming the population inversion in optical pumping laser systems. Change of energy spectrum with laser field provides a new freedom degree in optical systems based on interband and intersub-band transitions and also important advantage in the field of application.

**Figure 3:**
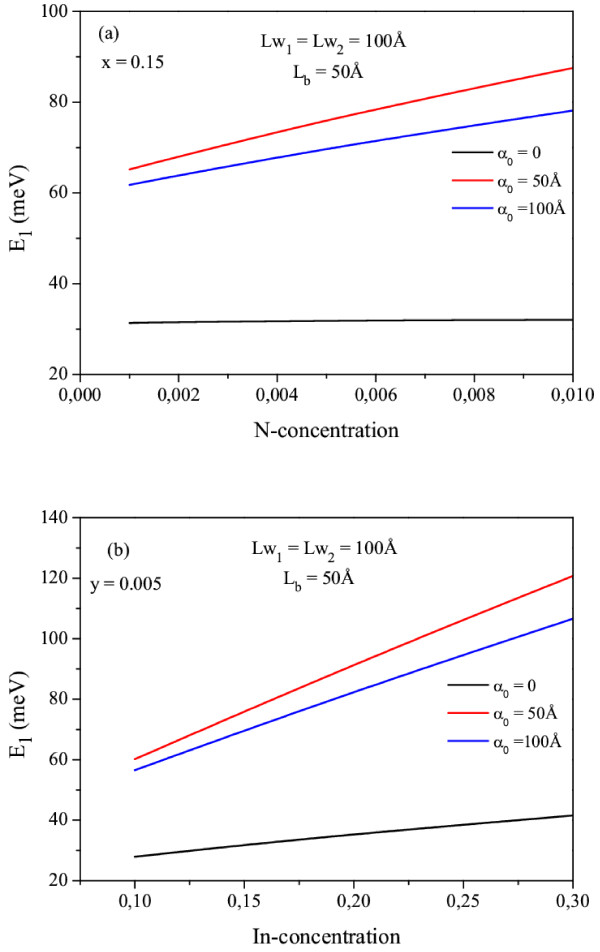
**Change of ground state energy levels.** As a function of N (**a**) and In (**b**) concentrations in Ga_1 − *x*_In_*x*_N_*y*_As_1 − *y*_/GaAsDQW for different laser-dressing parameters.

In Figure 3a,b, we display the change of ground state energy levels in Ga_1 − *x*_In_*x*_N_*y*_As_1 − *y*_/GaAs DQW for different laser-dressing parameters as a function of the N and In concentrations, respectively. As can be seen in this figure, as the N (In) concentration increases, the ground state energy levels increase. The main reason for this behavior is that for a constant In concentration, as N concentration increases, both the electron effective mass and the conduction band offset increase. Furthermore, the conduction band offset increases while the electron effective mass decreases with increasing In concentration for a constant N concentration. Additionally, the ground state energy level increases up to the certain laser value (*α*_0_ = 50 and 125 Å). On the contrary, it decreases when the laser field is further increased (see Figure 2).

## Conclusions

In this work, we have investigated mainly the effects of the ILF, N, and In concentrations on the bound states in Ga_1 − *x*_In_*x*_N_*y*_As_1 − *y*_/GaAs DQW. The calculations were performed within the effective-mass and envelope-wave function approximations. The frequency and corresponding laser intensity for *α*_0_ = 150 Å are 30 THz and 1.8 × 10^10^ W/cm^2^, respectively. The corresponding applied field intensity is the order of the crystal damage threshold intensity that can be avoided by using high-power pulsed CO_2_ lasers, etc. Fortunately, the current generation of free electron lasers can provide intense laser fields in the frequency range of 0.2 to 3,226 THz, with field strengths up to approximately 100 kV/cm. Therefore, our results can be tested by using the applied field intensity lower than the breakdown limit of the corresponding semiconductors.

Our numerical results reveal that ILF creates an additional geometric confinement on the electronic states in the DQW; the effect of the N (In) concentration on the electronic states increases with the effect of ILF. We can tune the electronic structure and main optical properties of the system which depend on intersub-band transitions by changing the N (In) concentration together with the laser field. We hope that our calculation results can stimulate further investigations of the related physics as well as device applications of dilute nitrides.

## Competing interests

The authors declare that they have no competing interests.

## Authors’ contributions

IS and MCA defined the theoretical framework of the study. FU, UY, and SS conducted the numerical calculations, prepared the computer programs, and gathered the research data. AE, EK, and HS analyzed the data findings and contributed to the conclusions. All authors read and approved the final manuscript.

## References

[B1] Reed MA Quantum dots Sci Am 1993 268 118

[B2] Loss D DiVicenzo DP Quantum computation with quantum dots Phys Rev A 1998 57 120 10.1103/PhysRevA.57.120

[B3] Jiang X Li SS Tidrow MZ Study of intersubband transition in quantum dots and quantum dot infrared photodetectors Physica E 1999 5 27 10.1016/S1386-9477(99)00026-0

[B4] Kristaedter N Schmidt OG Ledentsov NN Bimberg D Ustinov VM Yu A Zhukov AE Maximov MV Kopev PS Alferov ZI Gain and differential gain of single layer InAs/GaAs quantum dot injection lasers Appl Phys Lett 1996 69 1226 10.1063/1.117419

[B5] Imamura K Sugiyama Y Nakata Y Muto S Yokoyama N New optical memory structure using self-assembled InAs quantum dots Jpn J Appl Phys 1995 34 L1445 10.1143/JJAP.34.L1445

[B6] Kondow M Uomi K Niwa A Kitatani T Watahiki S Yazawa Y GaInNAs: a novel material for long-wavelength-range laser diodes with excellent high-temperature performance Jpn J Appl Phys 1996 35 1273 10.1143/JJAP.35.1273

[B7] Kondow M Kitatani T Nakahara K Tanaka T A 1.3 μm GaInNAs laser diode with lifetime of over 1000 hours Jpn J Appl Phys 1999 38 L1355 10.1143/JJAP.38.L1355

[B8] Kitatani T Nakahara K Kondow M Uomi K Tanaka T A 1.3 μm GaInNAs/GaAs single-quantum well laser diode with high characteristic temperature over 200 K Jpn J Appl Phys 2000 39 L86 10.1143/JJAP.39.L86

[B9] Tansu N Yeh JH Mawst LJ Low-threshold 1317-nm InGaAsN quantum-well lasers with GaAsN barriers Appl Phys Lett 2003 83 2512 10.1063/1.1613998

[B10] Gonul B Oduncuoglu M Dindaroglu S Yagdiran B Influence of doping on gain characteristics of GaInNAs/GaAs quantum well lasers Semicond Sci Technol 2003 18 163 10.1088/0268-1242/18/2/318

[B11] Galluppi M Geelhaar L Reichert H Nitrogen and indium dependence of the band offsets in InGaAsN quantum wells App. Phys Lett 2005 86 131925 10.1063/1.1898441

[B12] Tansu N Quandt A Kanskar M Mulhearn W Mawst LJ High-performance and high-temperature continuous-wave-operation 1300 nm InGaAsN quantum well lasers by organometallic vapor phase epitaxy Appl PhysLett 2003 83 18

[B13] Kondow M Kitatani T Nakatsuka S Larson MC Nakahara K Yazawa Y Okai M GaInNAs: a novel material for long-wavelength semiconductor lasers IEEE J Sel Top Quantum Electron 1997 3 719 10.1109/2944.640627

[B14] Lyngnes O Berger JD Prineas JP Park S Khigrova G Jahnke F Gibbs HM Kira M Koch SW Nonlinear emission dynamics from semiconductor microcavities in the nonperturbative regime Solid State Commun 1997 104 297 10.1016/S0038-1098(97)00279-2

[B15] Quochi F Bongiovanni G Mura A Staehli JL Deveaud B Stanley RP Oesterle U Houdre R Strongly driven semiconductor microcavities: from the polariton doublet to an ac stark triplet Phys Rev Lett 1998 80 4733 10.1103/PhysRevLett.80.4733

[B16] Quochi F Ciuti C Bongiovanni G Mura A Saba M Oesterle U Depertuis MA Staehli JL Deveaud B Strong coherent gain from semiconductor microcavities in the regime of excitonic saturation Phys Rev B 1999 59 R15594 10.1103/PhysRevB.59.R15594

[B17] Miranda LCM Energy-gap distortion in solids under intense laser fields Solid State Commun 1983 45 783 10.1016/0038-1098(83)90799-8

[B18] Nunes OAC Parametric distortion of the optical absortion edge of a magnetic semiconductor by a strong laser field J Appl Phys 1985 58 2102 10.1063/1.335974

[B19] Gerk E Miranda LCM Quantum well lasers by long wavelength radiation Appl Phys Lett 1984 44 837 10.1063/1.94942

[B20] Peyhambarian N Koch SW Lindberg M Fluegel B Joffre M Dynamic Stark effect of exciton and continuum states in CdS Phys Rev Lett 1989 62 1185 10.1103/PhysRevLett.62.1185 10039598

[B21] Enders BG Lima FMS Nunes OAC Fonseca ALA Agrello DA Qu F Da Silva EF Jr Freire VN Electronic properties of a quasi-two-dimensional electron gas in semiconductor quantum wells under intense laser fields Phys Rev B 2004 70 035307

[B22] Burileanu LM Niculescu EC Esanu N Radu A Polarizabilities of shallow donors in inverse V-shaped quantum wells under laser field Physica E 2009 41 856 10.1016/j.physe.2009.01.008

[B23] Ungan F Yesilgul U Sakiroglu S Kasapoglu E Sari H Sökmen I Effects of an intense, high-frequency laser field on the intersubband transitions and impurity binding energy in semiconductor quantum wells PhysLettA 2010 374 2980

[B24] Varshni YP Effect of an intense laser field on donor impurities in spherical quantum dots Superlatt Microstruct 2001 30 45 10.1006/spmi.2001.0992

[B25] Radu A Niculescu EC Cristea M Laser dressing effects on the energy spectra in different shaped quantum wells under an applied electric field J Optoelectron Adv Mater 2008 10 2555

[B26] DinizNeto OO Qu F Effects of an intense laser field radiation on the optical properties of semiconductor quantum wells Superlatt Microstruct 2004 35 1 10.1016/j.spmi.2004.05.004

[B27] Lima FMS Amato MA Nunes OAC Fonseca ALA Enders BG Da Silva EF Unexpected transition from single to double quantum well potential induced by intense laser fields in a semiconductor quantum well J Appl Phys 2009 105 123111 10.1063/1.3153963

[B28] Gavrila M Kaminski JZ Free-free transitions in intense high-frequency laser fields Phys Rev Lett 1984 52 613 10.1103/PhysRevLett.52.613

[B29] Pont M Walet NR Gavrila M McCurdy CW Dichotomy of the hydrogen atom in superintense, high-frequency laser fields Phys Rev Lett 1988 61 939 10.1103/PhysRevLett.61.939 10039473

[B30] Kramers H Collected Scientific Papers 1956 Amsterdam: North-Holland

[B31] Gavrila M Atoms in Intense Laser Fields 1992 Boston: Academic Press

[B32] Qu F Fonseca ALA Nunes OAC Hydrogenic impurities in a quantum well wire in intense, high-frequency laser fields Phys Rev B 1996 54 16405 10.1103/PhysRevB.54.16405 9985755

[B33] Kasapoglu E Duque CA Sari H Sökmen I Intense laser field effects on the linear and nonlinear intersubband optical properties of a semi-parabolic quantum well Eur Phys J B 2011 82 13 10.1140/epjb/e2011-20243-6

[B34] Kasapoglu E Sökmen I Shallow donor impurity binding energy in the V-shaped quantum well under the crossed electric and magnetic fields Physica E 2005 27 198 10.1016/j.physe.2004.11.002

[B35] Ng ST Fan WF Dang YX Yoon SF Comparison of electronic band structure and optical transparency conditions of In_x_Ga_1−x_As_1−y_N_y_∕GaAs quantum wells calculated by 10-band, 8-band, and 6-band k·p models Phys Rev B 2005 72 115341

[B36] Chuang SL Physics of Optoelectronic Devices 1995 New York: Wiley 709

[B37] Wu J Shan W Walukiewicz W Band anticrossing in highly mismatched III-V semiconductor alloys Semicond Sci Technol 2002 17 860 10.1088/0268-1242/17/8/315

[B38] Skierbiszewski C Experimental studies of the conduction-band structure of GaInNAs alloys Semicond Sci Technol 2002 17 803 10.1088/0268-1242/17/8/309

